# Alarm bells ring: suicide among Chinese physicians: A STROBE compliant study

**DOI:** 10.1097/MD.0000000000007790

**Published:** 2017-08-11

**Authors:** Yu Wang, Lei Liu, Hongbin Xu

**Affiliations:** a Hospital Development Center of Liaoning Province; b Department of Ophthalmology, The First Affiliated Hospital of China Medical University, Shenyang, China.

**Keywords:** physician, stress, suicide

## Abstract

To our surprise, there were 2 Chinese doctors who committed suicide in the last 2 months. What are the reasons for more and more Chinese doctors to commit suicide?

We present 18 cases of China's physicians who committed suicide.

We summarized the main reasons for the suicide of Chinese doctors from 2004 to 2017, and the main causes included clinical working stress, Job title promotion requirements stress, tension between the doctors and patients, and shying away from necessary treatment of their illness.

It is a cruel fact that more and more physicians died of suicide in China. We hope that suicide among Chinese doctors could be recognized so that it could be concerned with the understanding of human societies.

## 1 Introduction

Suicide, a complex public health problem, is the main leading cause of death in World.^[1]^ The latest news reported that a well-known Chinese ophthalmologist committed suicide by jumping to her death on January 6, 2017.^[2]^ To our surprise, a Chinese adult anesthesia doctor also committed suicide on February 15, 2017.^[3]^ These 2 doctors committing suicide in such a short period of time deserved our full attention. It is a cruel fact that more and more physicians in China died by suicide. The Chinese Medical Doctor Association (CMDA) reported that there were 16 doctors who committed suicide from 2004 to 2014 and most of them were young doctors.^[4]^ What are the reasons for more and more Chinese doctors to commit suicide? Nearly 85% to 90% persons who kill themselves have been suffering from some type of mental disorders.^[5]^ The main psychiatric diagnoses among doctors who commit suicide are affective disorders with depression or bipolar disease. So far, there is no study on the suicide of China's physicians. To the best of my knowledge, this is the first work that concerns suicide among Chinese physicians and summarizes the main reasons.

## 2 Methods

There is no epidemiological data regarding suicide on Chinese healthcare workers. In addition, doctors have less previous suicide attempts than other people. Hence, we can only summarize the main reasons for the suicide of Chinese doctors based on the news.^[4]^

Ethical approval is not necessary for this case series report and written consent could not be obtained from the patients who had committed suicide.

## 3 Results

Since 2004, there have been 18 Chinese physicians who committed suicide. Of all 18 physicians who committed suicide, there were 5 doctors who committed suicide because of conflicts between doctors and patients. For example, a 53-year-old male patient died during hernia surgery. The members of the patient's family considered that the patient died because the anesthesiologist had transformed the local anesthesia to intubation anesthesia in this patient and then they had a contradiction with this 50-year-old anesthesiologist and accused him. This anesthesia doctor committed suicide finally, for he could not bear the humiliation from the family members of this patient.^[3]^ Moreover, 5 doctors committed suicide because suffering from serious illness (such as cancer and depression) and the other physicians committed suicide because of stress from overload work or life.

Although it cannot explore differences in characteristics according to statistics, the detail information on these suicides such as gender, age, and means used to commit suicide is shown in Table 1.

**Table 1:**
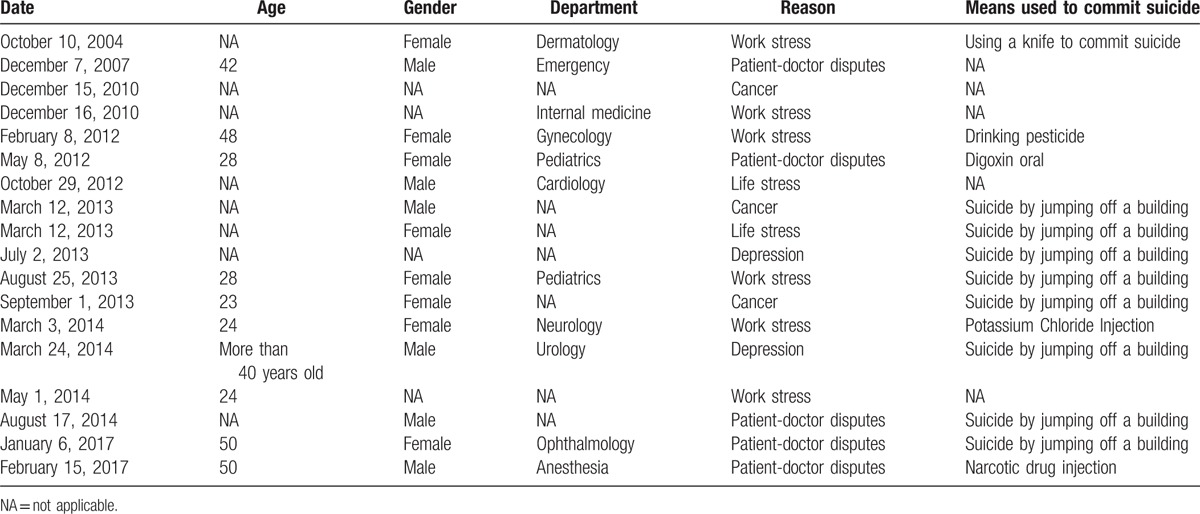
Characteristics for 18 China's physicians’ suicides.

## 4 Discussion

In our view, the main reasons for Chinese physicians’ suicide could be stress from the overburden of clinical work.^[6]^ Almost 70% of doctors work more than 50 hours per week and nearly 80% doctors work 8 to 12 hours per day in hospital. People working long hours are more likely to have a depression. There is a strong association between suicide and mental illnesses such as depression, drug dependence, and even borderline personality disorder (BPD).^[7]^ It was reported that there were nearly 91% suicide victims with mental illnesses.^[8]^ In many countries, depression is as common in the healthcare workers as in the general population, affecting an estimated 19.5% females and 12% males.^[9]^ What is more terrible is that 69.4% doctors showed signs of depression in Shanghai, China.^[4]^ Furthermore, 13.5% medical students had moderate-severe depression and 7.5% of them indicated the presence of suicidal ideation.^[10]^ Many doctors except psychologists are demonstrably poor at recognizing mental illnesses in patients, let alone themselves. There are many interventions for preventing physician mental illnesses and suicides have been taken in the United States. But in China, calls and interventions from Government are deficiency. We can imagine that the prevalence of suicide in physician would be increased with the clinical working stress increasing. It is needed to increase physicians’ awareness on indications of suicidal ideation, for example, some signs of serious depression.

Stress from research output-driven title promotion requirements. Job title promotion requirements by the government force Chinese doctors to apply for the Research Project Grant programs and publish articles indexed in the Science Citation Index (SCI). Owing to the heavy workload of clinical practice, some clinicians do not have the energy and interests to engage in research.^[11]^ At present, SCI articles are controlling the fate of China's physicians, which bring great pressure to the doctor, resulting in the psychological health on the edge.

The most important stress is from the current tensions between the doctors and patients in China.^[12]^ During 18 cases of suicides, nearly 30% of them could be attributed to the doctor–patient conflicts.^[4]^ Many doctors cannot tolerate “Yi Nao” problem and choose methods of suicide.^[2–4]^ Medical trouble has deteriorated into medical violence in China,^[13]^ and the violence against medical staff in hospital has been rising in recent years. No effective measures have been taken to prevent hospital violence so far. With disappointment, curse, even beating, many doctors choose methods of suicide in order to solve the conflicts between the doctor and the patient in China. How to end violence against doctors has become a serious problem in China.^[14]^

In addition, Chinese are always conservative, and Chinese doctors are more often ashamed to treat their own illnesses.^[4]^ Generally speaking, physicians believe that seeking treatment of illnesses for themselves may subject to stigma, shame, or worse. For those who are terminally ill, they prefer to suicide to end their lives without receiving treatments. Due to having more medical knowledge and better access to fatal measures, doctors have a higher prevalence of suicide attempts than the general population. Further epidemiological studies are needed to access adequate evidence for psychopathology among physicians who committed suicide in China.

## 5 Conclusion

In summary, we hope that suicide among Chinese doctors could be recognized by society, and it is necessary to launch an effective program to address this situation. After all, the doctors who are psychologically and physically healthy can better serve their patients when diagnosing them.

## Acknowledgments

The authors would like to thank Heather Hope in Sunbo Foreign Language School for language editing.
